# Alteration of Cytokine Profiles in Uranium Miners Exposed to Long-Term Low Dose Ionizing Radiation

**DOI:** 10.1155/2014/216408

**Published:** 2014-04-29

**Authors:** Kun Li, YiShui Chen, XiaoLiang Li, ShuJie Lei, QingFeng Chen, JianXiang Liu, QuanFu Sun

**Affiliations:** ^1^Key Laboratory of Radiological Protection and Nuclear Emergency, National Institute for Radiological Protection, Chinese Center for Disease Control and Prevention, 2 Xinkang Street, Deshengmenwai, Xicheng District, Beijing 100088, China; ^2^Institute of Occupational Medicine of Jiangxi, 336 Yongwaizheng Street, Nanchang 330006, China

## Abstract

*Objective.* The aim of the study is to estimate the immune function through cytokine profiles in sera of uranium mines. *Methods.* Antibody arrays were used to detect 50 cytokines in sera of uranium miners. Miners who had continuously worked underground for <5 years were treated as control group and those who worked for **⩾**5 years as experimental group. *Results.* Of 28 measurable cytokines, the release of IL-1**α**, IL-1RI, IL-15, IL-3, and IP-10 were significantly upregulated in the experimental group, and no cytokine was found significantly downregulated. Other proinflammatory cytokines such as IFN-*γ*, IL-10, IL-6, and TNF**α** levels were slightly upregulated in the experimental group. With adjustment to age, BMI, and cigarette smoking, IL-1**α** and IL-3 levels increased significantly with underground time. *Conclusion.* Alteration of cytokine profiles in this study may indicate persistent inflammatory responses in uranium miners exposed to long-term low doses radiation.

## 1. Introduction 

The immune-suppressing effect of high-dose radiation was clearly demonstrated and confirmed both in experimental and epidemiological studies [1, 2]. The effects of low dose radiation (LDR) on the immune system, on the other hand, both stimulatory and suppressive effects, have been reported by estimating changes in cell numbers or by using a variety of functional assays [3–8]. The long-term impacts of low radiation dose on the immune functions in relation to human health are controversial and need to be evaluated [9–12]. As a representative occupational subgroup, uranium mine workers are exposed to internal radiation mainly caused by radon and its progeny and external radiation from gamma radiation. External radiation represents 28% of total dose for underground miners [13], and, in China, gamma radiation dose that underground miners received was about 4 mSv/year based on the monitoring data gained in recent years [14]. It had been observed, in human populations inhabiting nearby a deactivated uranium mine, that immune functions were impaired by a significantly decreased NK and T lymphocytes counts [15]. However, the epidemiology study of uranium miners concerning immune function is scarce.

Cytokines, as the most important mediators by which cells of the immune system communicate, could be up- or downregulated by LDR [16–20]. However, the dysregulated expression of a special cytokine after irradiation does not sufficiently implicate its correlation with the pathogenesis [21]. Therefore, we estimated cytokine profiles to reflect the functional status of the immune system in this study. Furthermore, some cytokines in serum of healthy persons is generally low and no measurable level of cytokines was obtained using conventional ELISA (enzyme-linked immunosorbent assay) methods in preliminary experiments. Therefore, antibody microarray, having a higher sensitivity and greater detection range that promises to be a powerful tool for detecting multiple cytokine-expression levels simultaneously [22], was performed in this study to detect the relative expression of cytokine profiles. The aim of the present study is to estimate immune function with an emphasis on cytokine profiles in serum of workers from a uranium mine using antibody arrays.

## 2. Materials and Methods

### 2.1. Participants

We studied subjects from a uranium mine in China and classified the miners into two groups based on continuous underground time because of the lack of dose of workers exposed to uranium. The control group included 21 male persons who continuously worked underground for <5 years (cumulative dose <20 mSv, based on 4 mSv per year), and the experiment group included 28 male miners continuously working underground for *⩾*5 years (*⩾*20 mSv). On the day of blood sampling, all participants were subjected to medical examination and to routine haematological and biochemical tests for determination of their present health state, which revealed that they were basically healthy. This study obtained institutional approval from the human investigation committee and informed consents from participants.

### 2.2. Cytokines Analysis

Blood samples were collected from antecubital vein (between 7 a.m. and 9 a.m. before taking breakfast) of workers. Sera were obtained with blood centrifugation at 3600 r.p.m for 15 min and stored in a freezer at 4°C. Fifty cytokine assay kits were custom made using Human G-Series Array (RayBiotech, Inc., Norcross, GA); one antibody array slide includes 14 subarrays, and each subarray contains 50 different cytokines in duplicated spots. The relative concentrations of cytokines were detected according to the manufacturer's instructions. Briefly, wells of the microarray glass slides were blocked in blocking buffer at room temperature for 30 min and subsequently incubated with 100 *μ*L of 2-fold diluted sera overnight at 4°C. Slides were washed in washing buffer and incubated with a biotin-conjugated anticytokines for 2 h. After further washing, samples were incubated with 70 *μ*L of fluorescent dye conjugated per well in darkness for 2 h. Centrifuge at 1000 rpm for 3 min to remove water droplets. The images were captured using a LuxScan10K-A scanner. Spots signal intensities were imported into a RayBio antibody array tool for analysis automatically.

### 2.3. Statistical Analysis

The density of individual cytokines in all subjects was detected in duplicate. The average of the duplicate spots for each cytokine was normalized to the average of four positive controls on each array. The levels of cytokines in which the signal value of half the samples between two groups was above 200 were selected to further analysis. Group differences were analyzed with the SAM 3.00 algorithm. Any increase equal to or larger than 1.5-fold or decrease equal to or lesser than 0.65-fold in signal intensity for a single cytokine between the two groups is considered significant difference in expression. The significant difference is indicated by* q* value. Thereafter, the relationship between continuous mining time and the concentrations of measurable cytokines were assessed in total group of subjects using a multivariate linear regression model performed with STATA software (Texas, USA).

## 3. Results 

**Figure 1:**
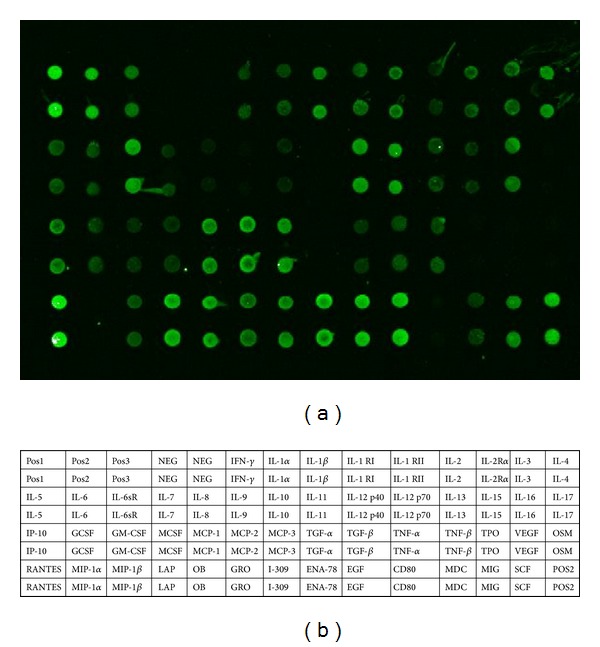
The cytokine profiles in sera of the subjects. (a) The comparative level of cytokines in sera of workers exposed to low dose irradiation were measured using Human G-Series Custom Array (RayBiotech, Inc., Norcross, USA) according to the manufacturer's suggestion. (b) The names of cytokines were list in the box.

Of the 50 custom cytokines, 28 cytokines above 200 signal value were suggestive of measurable cytokines (Figure 1). Compared to the control group, miners in the exposed group showed increased secretion in IL-1*α* by 1.712 fold, IL-1RI by 1.650 fold, IL-15 by 1.586 fold, IL-3 by 1.622 fold, and IP-10 by 1.767 fold and there was no cytokines significantly downregulated. Besides, expression of other proinflammatory cytokines, such as IFN-*γ*, IL-10, IL-6, and TNF*α*, was slightly upregulated in the exposed miners; the fold change is 1.220, 1.229, 1.246, and 1.172, respectively (Table 1).

In our study, age distribution, body mass index (BMI), and cigarette smoking were similar to each other in the exposed and control group (Table 2). We used multiple regression analysis to explore the relationships between the secretion levels of upregulated cytokines and important covariates like age, mining time, body mass index, and cigarette smoking. The release of IL-1*α* and IL-3 increased significantly with underground mining time with adjustment to age, BMI, and current smoking (Table 3). Conversely, age, BMI, and current smoking were not significantly related with the release of IL-1*α* and IL-3.

## 4. Discussion 

In this sera assay, immune responses of uranium miners were studied with the emphasis on cytokine-expression profiles. In our study, the levels of 28 cytokines measured in selected 50 cytokines showed that IL-1*α*, IL-1RI, IL-15, IL-3, and IP-10 levels were significantly upregulated in miners working for more than 5 years and there was no significantly downregulated cytokines between the two groups. With adjustment to age, BMI, and current smoking, IL-1*α* and IL-3 levels increased significantly with underground time.

The main biological activity of IL-1 is the stimulation of T-helper cells, which are induced to secrete IL-2 and to express IL-2 receptors. IL-1*α* may be a pathogenetic factor in the complex processes leading to vascular occlusion and an important in situ indicator of and a potential participant in vascular injury [23]. In this study, IL-1*α* concentration in sera of miners working underground for more than 5 years was significantly higher than those for lesser than 5 years, indicating the inflammatory response occurring with long-term exposure to LDR. Moreover, the linear relationship between the underground time and the level of IL-1*α* in sera of miners was revealed as a statistically significant positive correlation, implying that the inflammatory production could be attributed to long-term LDR. IL-1 had been reported to be a differentiation- and maturation-inducing agent for a variety of cells and also can serve as a signal that initiates radioprotective events in vivo [24, 25].

IL-3 is secreted by basophils and activated T cells to support the growth and differentiation of T cells from the bone marrow in an immune response and stimulates the differentiation of multipotent hematopoietic stem cells into myeloid progenitor cells. Elevation of IL-3 with underground time in miners working underground more than 5 years indicated the hormesis effect on hematopoietic system and activation of immune responses after chronic LDR. This result was similar to the study about stimulating effect of mice exposed to chronic low dose *γ*-irradiation (0.7 mGy/h for 11.9  days to reach a total dose of 0.2 Gy) on bone marrow hematopoietic progenitor cell proliferation and peripheral blood mobilization [26].

IFN-*γ*, IL-10, IL-6, and TNF-*α* coordinate the inflammatory response. In the present study, all these cytokines levels increased slightly but not significantly with underground time. Taking the results mentioned above, our study provided further evidence of persistent inflammatory response in persons exposed to LDR. Inflammatory cytokines expression in our results were consistent with the study of atomic bomb survivors, in which IFN-*γ*, IL-10, IL-6, and TNF-*α* increased expression providing evidence of persistent inflammatory responses in atomic bomb survivors more than 50 years after radiation exposure [15].

From cytokine profiles expressed in this study, we concluded that uranium miners receiving more than 20 mSv per year produced the persistent inflammatory responses. There is evidence that short-term low doses irradiation can induce anti-inflammatory effects through the secretion of modulatory cytokines. Inflammatory cytokines IL-1*β* and TNF-*α* releases were reduced in peritoneal macrophages of Balb/c mice responding to exposure of 0.5 or 0.7 Gy of ionizing irradiation (X-ray) [27]. Meanwhile, low/moderate doses (0.5 Gy or 1.0 Gy) exposure is clinically used to treat benign inflammatory diseases and is therefore capable of downregulating inflammation [28, 29]. However, in our study, it seems to suggest that inflammatory responses occurred after long-term exposure to low dose ionizing radiation and the mechanism between them is an area of current research. In addition, all the people we surveyed are healthy people and the concentrations of IL-1*α* and IL-3 are pretty low in normal serum, so we could not quantity the levels in this study. For further study, it appears necessary to ensure an adequate study and control population and to get larger sample sizes and verify the cytokines expression, which may lead to significant progress in our understanding of the effects of chronic, low dose radiation exposure on immune functions.

## 5. Conclusion

Our study provides the first evidence to show cytokine profiles in sera of uranium miners and explore immune function after long-term low dose of ionizing radiation. We hypothesize that modification of cytokine production may indicate persistent inflammatory responses in uranium miners working underground for more than 5 years and cumulative dose is about 20 mSv. The emphasis of our next work is to ensure an adequate population to get large sample sizes and verify the cytokines expression in human populations exposed to long-term low dose of ionizing radiation.

## Figures and Tables

**Table 1:** Twenty-eight cytokines measurable in serum of uranium miners.

Cytokine	Fold change	*q* value (%)
IP-10	1.767	<0.05
IL-1*α*	1.712	<0.05
IL-1sRI	1.650	<0.05
IL-3	1.622	<0.05
IL-15	1.586	<0.05
IL-2	1.427	<0.05
GM-CSF	1.404	<0.05
IL-13	1.361	<0.05
TNF-*β*	1.357	<0.05
IL-2sR*α*	1.315	3.1
IL-7	1.288	<0.05
MCP-2	1.278	3.1
IL-6	1.246	3.1
ENA-78	1.237	3.1
GRO	1.232	3.1
IL-10	1.229	<0.05
GCSF	1.227	3.1
IFN-*γ*	1.220	3.1
TGF-*β*	1.213	<0.05
TNF-*α*	1.172	3.1
MIG	1.153	3.1
EGF	1.085	3.1
MIP-1*β*	1.065	3.1
LAP	1.008	3.1
IL-8	0.987	>5
RANTES	0.966	>5
MCP-1	0.934	>5
IL-6sR	0.894	>5

**Table 2:** Characteristics of the study subjects.

Characteristic	Control group working for <5 years (*n* = 21)	Experimental group working for >5 years (*n* = 28)
	Number (%) or mean ± SD	
Age	45.04 ± 4.86	47.36 ± 2.91
BMI	24.26 ± 3.54	23.64 ± 2.93
Current smokers	10 (47.62)	16 (57.14)

**Table 3:** Multivariate models of the effects of underground time and age on IL-1*α* and IL-3 expression.

Variable	IL-1*α*	IL-3
	Percentage Increment (95% Confidence Interval)	
Time per 10 years	30 (9–51)	38 (6–70)
Age per 10 years	−27 (−9–35)	−78 (−173–17)
